# Integrating clinical and cross-cohort metagenomic features: a stable and non-invasive colorectal cancer and adenoma diagnostic model

**DOI:** 10.3389/fmolb.2023.1298679

**Published:** 2024-01-22

**Authors:** Dan Zhou, Youli Chen, Zehao Wang, Siran Zhu, Lei Zhang, Jun Song, Tao Bai, Xiaohua Hou

**Affiliations:** ^1^ Division of Gastroenterology, Union Hospital, Tongji Medical College Medical College, Huazhong University of Science and Technology, Wuhan, China; ^2^ State Key Laboratory for Oncogenes and Related Genes, NHC Key Laboratory of Digestive Diseases, Division of Gastroenterology and Hepatology, Shanghai Institute of Digestive Disease, Renji Hospital, School of Medicine, Shanghai Jiao Tong University, Shanghai, China; ^3^ School of Management, Huazhong University of Science and Technology, Wuhan, China

**Keywords:** colorectal cancer, colorectal adenoma, machine learning, gut microbiome, metagenomics

## Abstract

**Background:** Dysbiosis is associated with colorectal cancer (CRC) and adenomas (CRA). However, the robustness of diagnostic models based on microbial signatures in multiple cohorts remains unsatisfactory.

**Materials and Methods:** In this study, we used machine learning models to screen metagenomic signatures from the respective cross-cohort datasets of CRC and CRA (selected from CuratedMetagenomicData, each disease included 4 datasets). Then select a CRC and CRA data set from the CuratedMetagenomicData database and meet the requirements of having both metagenomic data and clinical data. This data set will be used to verify the inference that integrating clinical features can improve the performance of microbial disease prediction models.

**Results:** After repeated verification, we selected 20 metagenomic features that performed well and were stably expressed within cross-cohorts to represent the diagnostic role of bacterial communities in CRC/CRA. The performance of the selected cross-cohort metagenomic features was stable for multi-regional and multi-ethnic populations (CRC, AUC: 0.817–0.867; CRA, AUC: 0.766–0.833). After clinical feature combination, AUC of our integrated CRC diagnostic model reached 0.939 (95% CI: 0.932–0.947, NRI=30%), and that of the CRA integrated model reached 0.925 (95%CI: 0.917–0.935, NRI=18%).

**Conclusion:** In conclusion, the integrated model performed significantly better than single microbiome or clinical feature models in all cohorts. Integrating cross-cohort common discriminative microbial features with clinical features could help construct stable diagnostic models for early non-invasive screening for CRC and CRA.

## 1 Introduction

Colorectal cancer (CRC) ranks as the third most diagnosed cancer and a leading cause of death in both men and women globally (Sung et al., 2021). Colonoscopy is now considered the reference standard for the detection and prevention of CRC (Dekker and Rex, 2018). However, in clinical practice, factors such as unsatisfactory bowel preparation, invasiveness, long time and high expenses of examinations and appointments, etc., greatly limited the screening efficiency and patient adherence (Lee et al., 2012). In addition, colonoscopy is not suitable for the elderly or patients with contraindications. Therefore, a non-invasive tool is urgently needed for effective population-wide screening to optimize CRC prevention and diagnosis.

Up till today, various novel screening methods have been made available, including guaiac-based fecal occult blood tests (gFOBTs), fecal immunochemical tests (FIT), and newer non-invasive tests (e.g., blood or stool tests for DNA, RNA, and protein organisms’ markers), but their diagnostic performances and clinical value are yet unsatisfactory (Wong and Yu, 2019). As the precursor of CRC, colorectal adenomas (CRA) are particularly hard to detect using non-invasive methods (Imperiale et al., 2014). The research results of Niedermaier T et al. showed that the predictive sensitivity of FIT and DNA markers for advanced adenoma hardly exceeds 70% (Niedermaier et al., 2018).

Emerging evidence shows that CRC and CRA were accompanied by dysbiosis of the gut microbiome (Yu et al., 2017). For example, *Bacteroides* (e.g., *Bacteroides fragilis*) and a strain of *Escherichia coli* are closely related to colorectal carcinogenesis (Cuevas-Ramos et al., 2010; Arthur et al., 2012). Therefore, there has been emerging research focusing on the diagnostic value of gut microbiome for CRC and CRA(10).

However, several challenges still exist in establishing sound microbiome-based diagnostic models. Firstly, the composition of the intestinal flora varied among different regions and ethnic groups (Dwiyanto et al., 2021). To date, most studies are single-centered, and the integration of data from multiple populations is rare (Kim et al., 2020; Chen et al., 2022; Coker et al., 2022). Thus, common cross-cohort microbiome features remain unexplored. Secondly, due to the high dimensionality and redundancy of microbiome data generated by high-throughput sequencing methods, improving data processing pipelines for better utilization of microbiome information is crucial for model judgment, which requires multi-disciplinary proficiencies. Thirdly, it should be noted that CRA and CRC occurrence and development are affected by multiple factors. While multi-modality models integrating microbiota and serum metabolites have shown good performance (Chen et al., 2022; Gao et al., 2022), clinical and demographic data, which are accessible and have been proven as important risk factors (Song et al., 2020) have not yet been involved in integrated models for diagnosing CRA and CRC.

Machine learning (ML) algorithms are valuable candidates for fast and deep processing of high-throughput data, including metagenomic data (Cammarota et al., 2020). Additionally, these models could be trained to explore dynamic trends, such as common features across different regions and ethnics. In the field of IBD and liver disease, ML technology has been used to analyze large-scale data from different settings (e.g., demographic, laboratory and sequencing data), combine them and tap the potential for diagnostic prediction (Cammarota et al., 2020; Seyed Tabib et al., 2020; Liu et al., 2022).

**FIGURE 1:**
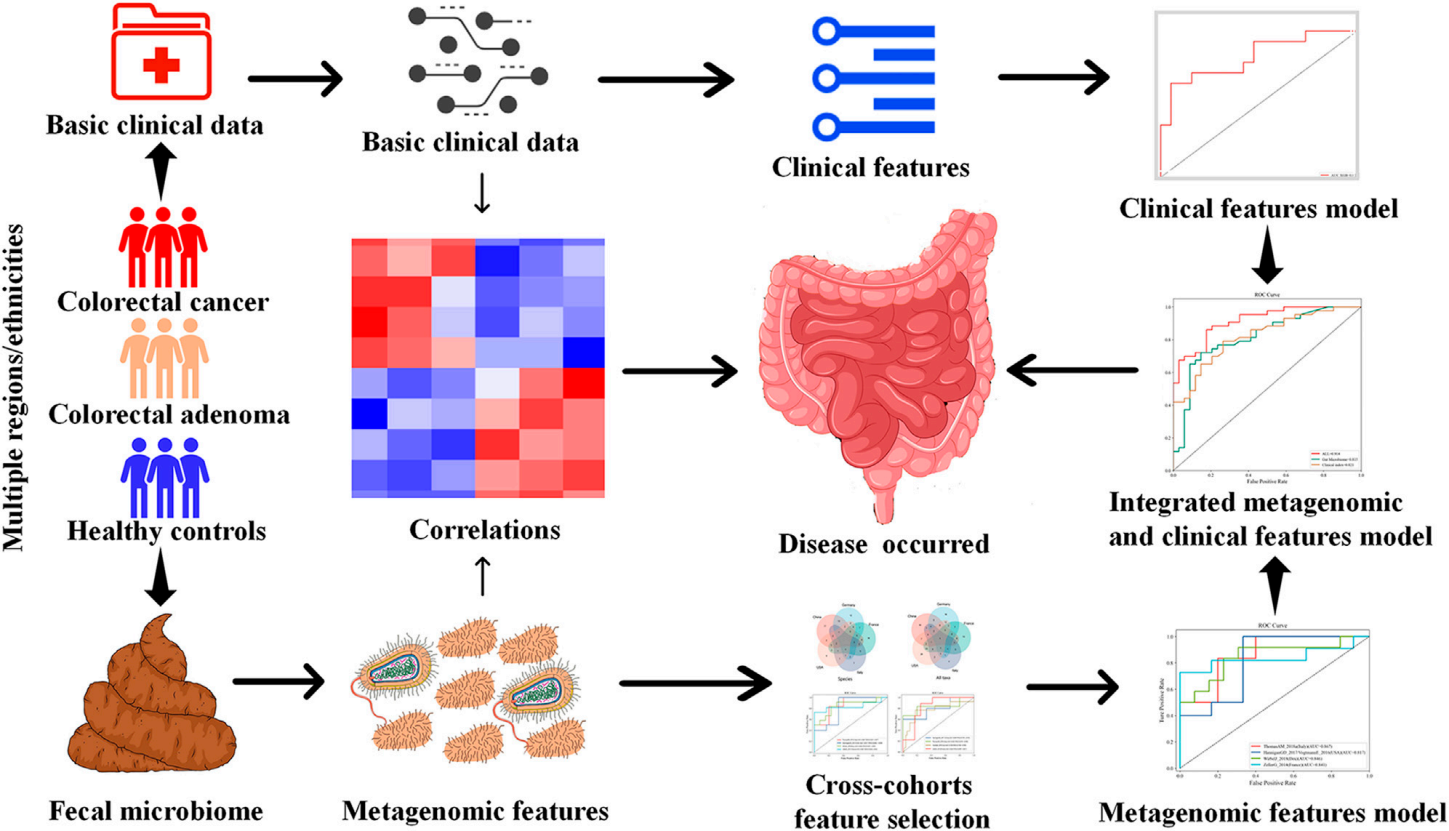
Study design.

Therefore, using multi-modal ML algorithms integrating demographic, clinical, and microbiome features, this study aims to screen for stable CRC and CRA-related metagenomic features in multiple cohorts, and establish robust prediction models for CRC and CRA (Figure 1).

## 2 Materials and methods

### 2.1 Study design and data preparation

Our purpose is to train a stable and reliable CRC or CRA disease diagnosis model that integrates microbial and clinical indicators, so we selected some datasets from a publicly available and standardized microbiome data sourcedatabase which name is MetagenomicData, and curated through BioConductor (Pasolli et al., 2017) that have both metagenomic and clinical data(see Table 1 for basic information on the data sets). Study subjects included stool samples from patients with colorectal cancer (CRC) or colorectal adenoma (CRA) and healthy controls (HC).

**TABLE 1:** Basic information of the CRC or CRA cross-cohort datasets.

#DEEAF6; ">	#DEEAF6; ">	#DEEAF6; ">	#DEEAF6; ">
Region	Sample size	Study name in curated MetagenomicData	PMID
CRC			
France^a^	HC = 61 CRC = 53	ZellerG_2014	25432777
United States^a^	HC = 52 CRC = 58	VogtmannE_2016	27,171,425
Italy^a^	HC = 24 CRC = 29	ThomasAM_2018a	30,936,548
Germany^a^	HC = 65 CRC = 60	WirbelJ_2018	30,936,547
China^b^	HC = 53 CRC = 75	YuJ_2017	26,408,641
CRA			
China and USA^c^	HC = 28 CRA = 26	HanniganGD_2017	30,459,201
Italy^c^	HC = 24 CRA = 27	ThomasAM_2018a	30,936,548
Japan^c^	HC = 251 CRA = 67	YachidaS_2019	31,171,880
France^c^	HC = 61 CRA = 42	ZellerG_2014	25432777
Australia^b^	HC = 61 CRA = 47	FengQ_2015	25,758,642

^a^Cross-cohort datasets included in the CRC, feature screening process.

^b^T:he dataset which used in the final integrated model.

^c^ Cross-cohort datasets included in the CRA, feature screening process.

We first analyzed and discussed the model methods and metagenomic data input methods in order to find the best way to maximize the value of multidimensional data. Secondly, we screened and verified multiple times in cross-cohorts data sets (Hereinafter referred to as the cross-cohorts data set, for CRC, it refers to: ZellerG_2014, VogtmannE_2016, ThomasAM_2018, WirbelJ_2018 data set; for CRA, it refers to HanniganGD_2017, ThomasAM_2018, YachidaS_2019, ZellerG_2014 data set) from different countries and races, and selected the 20 most stable and specific metagenomic features. Finally, the YuJ_2017 and FengQ_2015 datasets were used to build a diagnostic model integrating metagenomic and clinical features.

China’s YuJ_2017 (Yu et al., 2017) was selected as the final dataset for CRC, while Australia’s FengQ_2015 (Feng et al., 2015) was chosen as the final dataset for CRA. These datasets were chosen because their clinical data appear to be more relevant to the disease based on previous studies of traditional risk factors for the disease.

The YuJ_2017 dataset contains metagenomic data of 53 cases of CRC and 75 healthy subjects, and also records some of their clinical indicators: age, sexual, body mass index (BMI), triglycerides, high-density lipoprotein (HDL), low-density lipoprotein (LDL), cholesterol, creatinine, fasting glucose, Estimated glomerular filtration rate(eGFR) and alanine transaminase (ALT). Only age, triglycerides, HDL and fasting glucose were significantly different between the two groups (*p* = 0.012; *p* = 0.020; *p* < 0.001; *p* < 0.001). The FengQ_2015 data set contains metagenomic data of 47 CRA patients and 61 healthy participants, and also counts age, gender, BMI, triglycerides, HDL, LDL and co-morbid disease (type 2 diabetes mellitus, hypertension, fatty liver). However, none of the clinical indicators were significantly different between the two groups (*p* > 0.05). Please see Supplementary Table S1 for clinical data information of other data sets.

Microbiota features with less than 0.01% mean relative abundance and less than 10% prevalence were excluded. All clinical data included in this study are routine clinical tests and have been shown to correlate with disease in previous studies (Chapelle et al., 2020).

### 2.2 Selection the optimal model method

**FIGURE 2:**
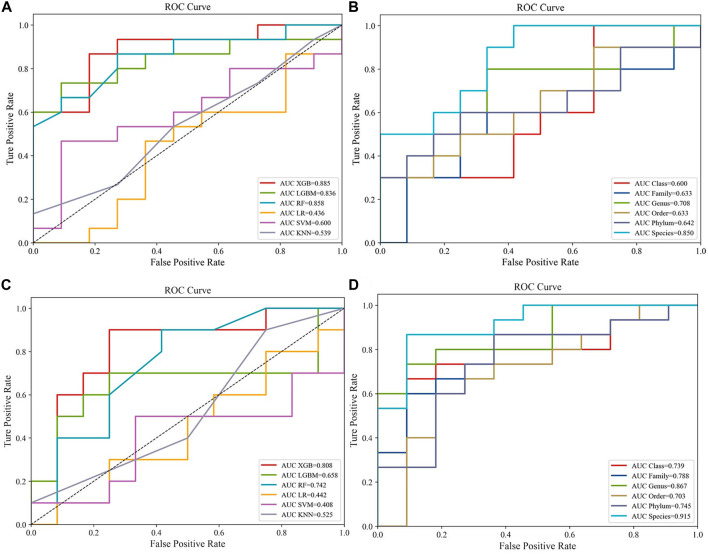
Compare the performance of different machine learning models:**(A)** Predictive performances of six machine learning methodologies [Extreme Gradient Boosting (Xgboost), Lightweight Gradient Boosting Machine (LGB), Random Forest (RF), Logistic Regression (LR), Support Vector Machine (SVM), k-nearest neighbors (KNN)] in the CRC dataset (YuJ_2017); **(B)** Predictive performances of single-level taxons (phylum, class, order, family, genus, species) in the CRC dataset (YuJ_2017); **(C)** Predictive performances of six machine learning methodologies [Extreme Gradient Boosting (Xgboost), Lightweight Gradient Boosting Machine (LGB), Random Forest (RF), Logistic Regression (LR), Support Vector Machine (SVM), k-nearest neighbors (KNN)] in the CRC dataset (YuJ_2017); **(D)** Predictive performances of single-level taxons (phylum, class, order, family, genus, species) in the CRA dataset (FQ_2015).

Popular ML methods, including Extreme Gradient Boosting (Xgboost), Lightweight Gradient Boosting Machine (LGB), Random Forest (RF), Logistic Regression (LR), Support Vector Machine (SVM), k-nearest neighbors (KNN) (Greener et al., 2022), were trialed for CRC/HC and CRA/HC classification. YuJ_2017 and FQ_2015 datasets were used as the CRC and CRA datasets to compare the machine learning model efficacie. Models were optimized by tuning hyperparameters and training. Hyperparameters are parameters that need to be specified or tuned by the user in order to train a model for a specific modeling problem(23) (Figure 2).

All models adopted the hold-out method, in which a dataset was divided into two mutually exclusive parts, one as the training set and the other as the test set (Siugzdaite et al., 2020). All models adopted the hold-out method, in which a dataset was divided into two mutually exclusive parts, one as the training set and the other as the test set (Siugzdaite et al., 2020). The ratio of samples in the training and test sets was 8:2. The ratios of cases and controls were also kept consistent in training and test sets to avoid biases introduced by the data partitioning process.

### 2.3 Selection of metagenomic features from cross-cohorts

**FIGURE 3:**
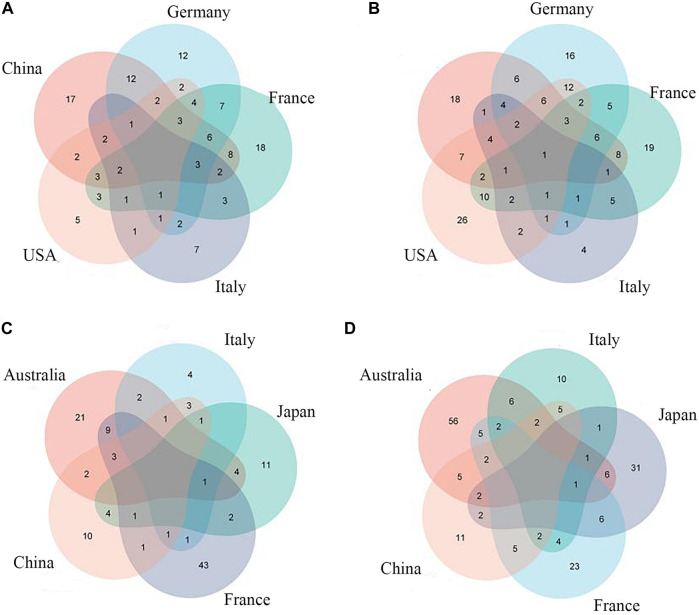
Common CRC/CRA microbial features across cohorts:**(A)** Species level (CRC); **(B)** Full taxonomic information (CRA); **(C)** Species level (CRA); **(D)** Full taxonomic information (CRA).

**FIGURE 4:**
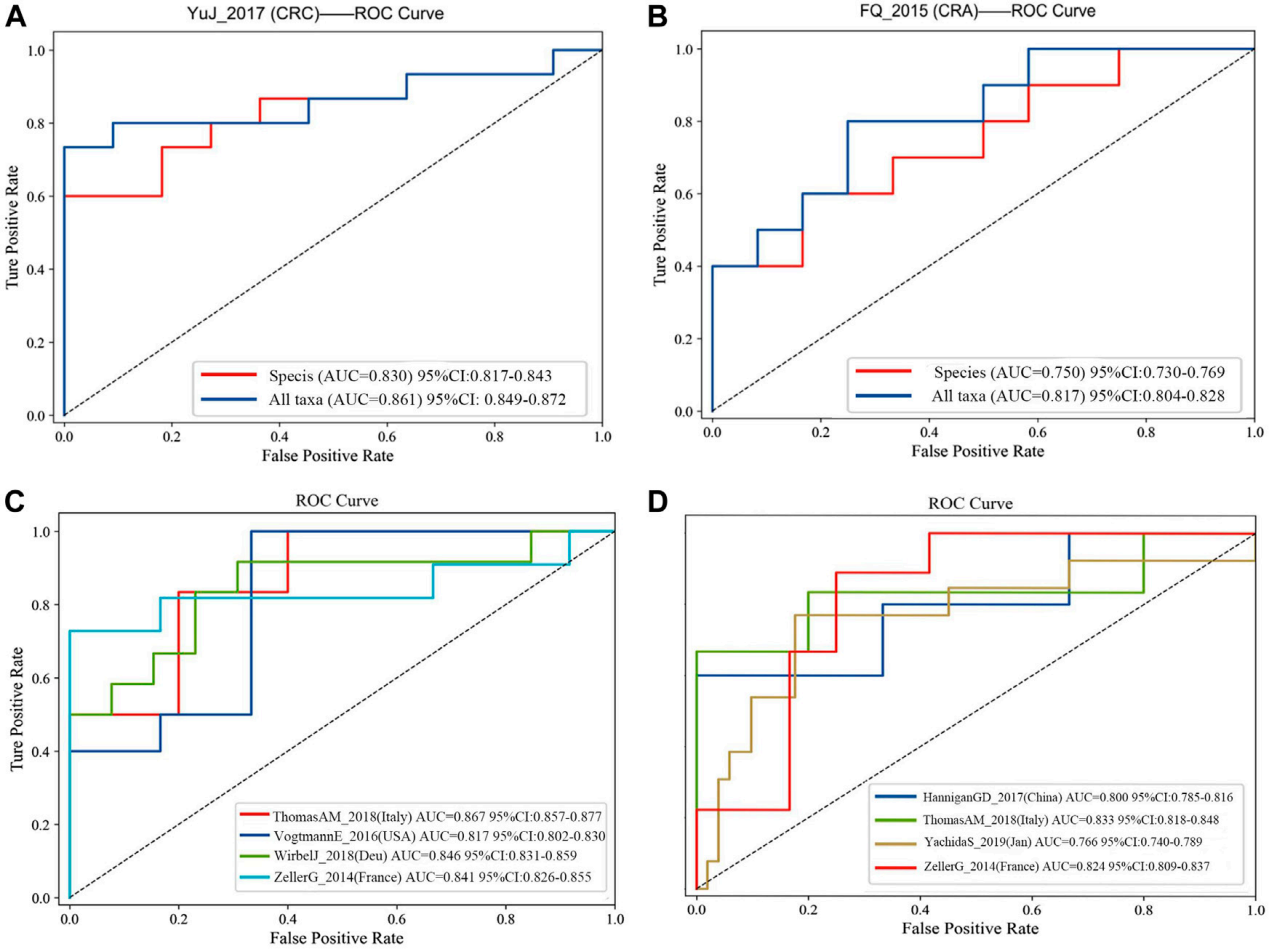
**(A)** Comparision of diagnostic efficacy in CRC dataset (YuJ_2017) using species-level information and full taxonomic information in the XGboost model; **(B)** Comparision of diagnostic efficacy in CRA dataset (FQ_2015) using species-level information and full taxonomic information in the XGboost model. Validation in multiple cohorts for diagnosis of CRC **(C)** and CRA **(D)** using all taxanomic information in XGboost model. AUC, area under curve. The dashed line represents AUC for 0.5. [CRC: ThomasAM_2018 (Italy) AUC 95% CI:0.857–0.977; VogtmannE_2016 (United States) AUC 95%CI: 0.802–0.830; WirbelJ_2018(Due) AUC 95% CI:0.831–0.859; ZellerG_2014 (France) AUC 95%CI: 0.826–0.855. CRA:HanniganGD_2017(China) AUC 95%CI:0.7850.816; ThomasAM_2018(Italy) AUC 95% CI:0.809–0.837].

Previous studies have shown that sensitivity and accuracy vary with the degree of clustering of metagenomic data when training for ML (Thomas et al., 2019). Therefore, we first input individual levels of taxonomic data (phylum, order, family, class, genus, species) into the model and selected the best-performing taxon level according to the area-under-the-curve (AUC) value of the model. After comparison, we found that it is easier to find the intersection of feature groups between different data sets by inputting all classification levels into the model for training (Figures 3, 4A,4B).

To improve the diagnositic value of metagenomic features in multi-regional and multi-ethnic datasets, we performed cross-cohorts validation during feature screening, and the feature groups that performed best in cross-cohorts were retained. The process was divided into two steps.

Firstly, all datasets selected for metagenomic feature screening (CRC: ZellerG_2014, HC = 61, CRC = 53; VogtmannE_2016, HC = 52, CRC = 52; ThomasAM_2018, HC = 24, CRC = 29; WirbelJ_2018, HC = 65, CRC = 60; CRA: HanniganGD_2017, HC = 28, CRA = 26; ThomasAM_2018, HC = 24, CRA = 27; YachidaS_2019,HC = 251, CRA = 67; ZellerG_2014, HC = 61, CRA = 42) were trained with disease diagnosis models using the XGboost method, with a training set and validation set ratio of 8 to 2, and performed SHAP analysis *post hoc*. Secondly, based on the results of SHAP analysis, we take the intersection of the features that contribute to disease judgment in each dataset (Feature value > 0). And use the filtered intersection features to verify again in the cross-cohorts data set. After multiple intersections, selection, and verification, and combined with previous relevant research bases, we finally obtained 20 metagenomic features. Their performance is stable in four cross-cohort data sets, with an average AUC value greater than 0.8 (Figures 4C,D).

**FIGURE 5:**
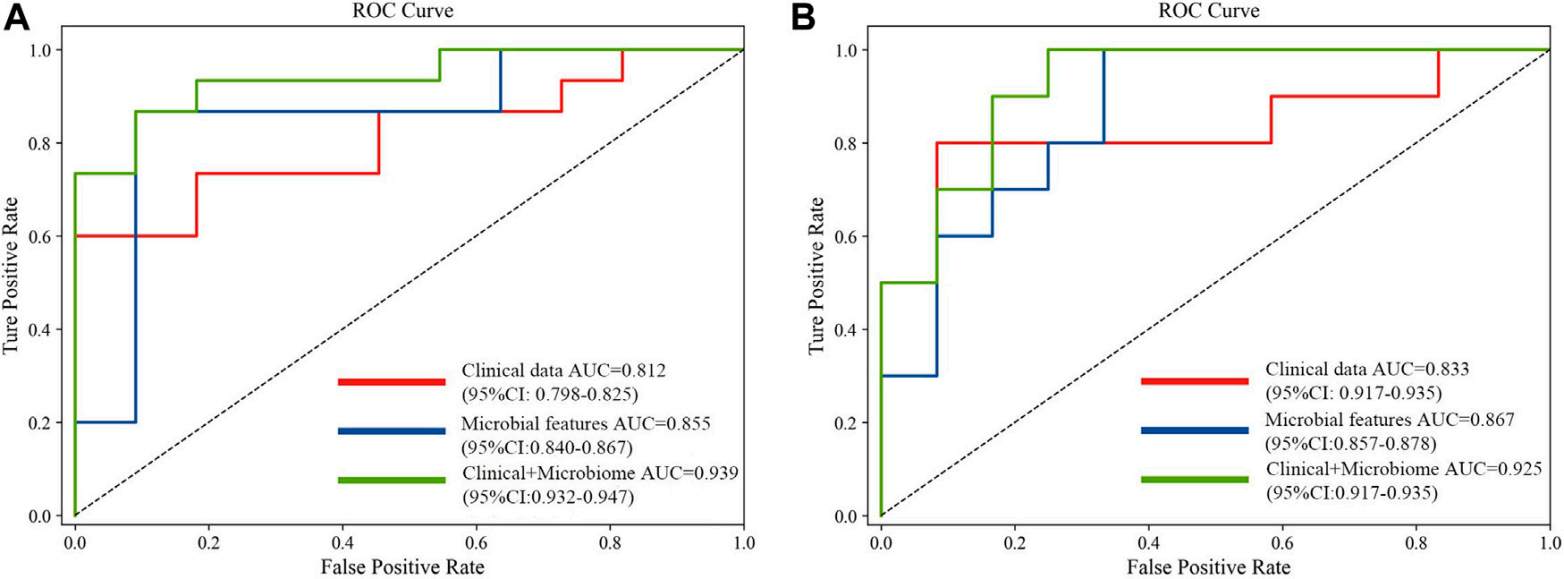
Comparison of approaches for the prediction of CRC **(A)** and CRA **(B)** using only clinical and demographical features, only microbial features, and the integrated method.

The 20 screened metagenomic features of CRC/CRA have good diagnostic results in the YuJ_2017 and FengQ_2015 data sets respectively, with AUC values of 0.855 (95%CI: 0.840–0.867) and 0.867 (95%CI: 0.857–0.878) respectively (Figure 5).

### 2.4 SHapley additive exPlanation (SHAP) analysis

SHAP analysis evaluates importance of a taxon using “Information gain (IG)”. That is, the ratio of each individual feature to the sum of features gets the score, and the average reduction of loss of the input features when used as a dividing attribute (the information gain of the features) (Kent, 1983; Wang et al., 2005; Gao et al., 2017; Lundberg and Lee, 2017).

IG g (Y, X) indicates the reduction of uncertainty as define below

$$G\left(Y,X\right)=H\left(Y\right)–H\left(Y|X\right)$$

where H(Y) denotes the entropy of dataset Y, which quantifies the uncertainty involved in predicting the value of a random variable, whereas H(Y|X) denotes the conditional entropy, which represents the uncertainty based on the known variable X. p denotes probability distribution. H(Y) and H(Y|X) are defined as follows:

$$H\left(Y\right)=–\sum p\left(y\right)\log p\left(y\right)$$

$$H\left(Y|X\right)=\sum_{x\in XP}\left(x\right)H\left(Y|X=x\right)$$

In this study, we used the Python 3.8 program language, the compiler version was PyCharm Community Edition 2021.1.1 x64. Among the ML models used in this paper, the LR, KNN, SVM and RF models were built using the scikit-learn package, the model XGboost was built using the Xgboost package, and the LGB model was built using the lightgbm package.

### 2.5 Construction of the integrated model of clinical and metagenomic features

We input the metagenomic and clinical features with stable performance in cross-cohorts together into the Xgboost model, and used AUC values for performance evaluation. External validation of this methodology was performed across multiple cohorts.

Xgboost can accept predictions in the absence of indicators, which is more adaptable to complex situations in practical applications. However, this study still used multiple imputation to fill in clinical missing values, because it ensured that subsequent analysis such as shap would be added to facilitate observation and discussion. Models were optimized by tuning hyperparameters and training. Hyperparameters are parameters that need to be specified or tuned by the user in order to train a model for a specific modeling problem (Topçuoğlu et al., 2020). In order to evaluate the performance of the model and prevent overfitting, 10-fold cross validation was introduced when building the model. The ratio of training set to test set for all models in this study is 8:2.

### 2.6 Statistical analysis

Statistical analysis was performed using R Statistical Software (version 4.1.2). Count data were described by frequency and composition ratios, while continuous data were expressed as median ± IQR, Normality was gauged using Kolmogorov-Smirnov tests. Differences between groups were compared by Chi-square and Mann-Whitney U tests. Correlations between groups were assessed using Spearman correlation analysis. A *p*-value≤ 0.05 for two-sided test or a *p*-value ≤0.025 for one-sided test is considered statistically significant.

## 3 Results

### 3.1 Clinical characteristics of participants

The previous section has provided a comprehensive description of the basic clinical information of each study participant. For further details, please refer to Supplementary Table S1.

### 3.2 Selection the optimal model method

XGBoost outperformed other machine learning model, and was used for model construction in this study (CRC, AUC: 0·875–0·895; CRA, AUC: 0·790–0·821) (Figures 2A,C).

### 3.3 Selection of metagenomic features from cross-cohorts

Species-level data performed best in single-level taxonomic (CRC, AUC: 0·906–0·922; CRA, AUC: 0·839–0·862) (Figures 2B,D), while all-level taxonomic data performed even better (CRC, AUC: 0·849–0·872; CRA, AUC: 0·804–0·828) (Figures 4A,B). And as shown in Figure 3, when only species-level metagenomic data was input, the intersection of feature values between all data sets in each region is small; if all classification-level data were input into the model together, the intersection between feature values of each data set is obviously increased (*p* < 0.01) (Figure 3).

The final 20 metagenomic features were selected to be stable and efficient in the cross-chorts cohort: CRC, AUC: 0·817–0·867; CRA, AUC: 0·760–0·833]. CRC: ThomasAM_2018 (Italy) AUC = 0.867, 95% CI:0.857–0.977; VogtmannE_2016 (USA) AUC = 0.817, 95%CI: 0.802–0.830; WirbelJ_2018(Due) AUC = 0.846,95% CI:0.831–0.859; ZellerG_2014 (France) AUC = 0.841 95%CI: 0.826–0.855. CRA: HanniganGD_2017(China) AUC = 0.800, 95%CI:0.785–0.816; ThomasAM_2018(Italy) AUC = 0.833, 95% CI:0.809–0.837; YachidaS_2019 (Japanese) AUC = 0.766, 95%CI:0.740–0789; ZellerG_2014 (France) AUC = 0.824 95%CI: 0.809–0.837 (Figures 4C,D).

The 20 screened metagenomic features of CRC/CRA also have good diagnostic results in the YuJ_2017 and FengQ_2015 data sets respectively, with AUC values of 0.855 (95%CI: 0.840–0.867) and 0.867 (95%CI: 0.857–0.878) respectively (Figure 5).

### 3.4 Multi-modal diagnostic model integrating metagenomic and clinical features

The performance of the model integrating the metagenomic and clinical features was significantly better than models including only clinical or metagenomic data for both CRC (metagenomic data, AUC: 0.855, 95%CI: 0.840–0.867; clinical data, AUC: 0.812, 95%CI: 0.798–0.825; combined, AUC: 0.939, 95%CI: 0.932–0.947) and CRA (metagenomic data, AUC: 0.867, 95%CI: 0.857–0.878; clinical data, AUC: 0·833, 95%CI: 0.917–0.935; combined, AUC: 0.925, 95%CI: 0.917–0.935) (Figure 5). An net reclassification improvement (NRI) value greater than 0 means that the added feature values contribute to model judgment. For CRC (YuJ_2017), the NRI value of the model integrating clinical indicators was 30% compared with the model built using only metagenomic features. For CRA (FengQ_2015), the NRI value of the model integrating clinical indicators was 18% compared with the model built using only metagenomic features (He et al., 2022). Table 2 presents detailed model results.

**TABLE 2:** Comparison of the performance of CRC/CRA single data model and integrated model.

Model	AUC	95% CI	Sensitivity	Accuracy
CRC				
Only clinical data	0·812	0·798–0·825	0·776	0·769
Only metagenomic data	0·855	0·840–0·867	0·855	0·846
Integrated clinical and metagenomic features	0·939	0·932–0·947	0·888	0·885
CRA				
Only clinical data	0·833	0·917–0·935	0·858	0·864
Only metagenomic data	0·867	0·857–0·878	0·783	0·773
Integrated clinical and metagenomic features	0·925	0·917–0·935	0·876	0·864

Improvement of model performance by adding clinical features was also demonstrated in validation datasets (Supplementary Figure S1). More details are shown in Table 3.

**TABLE 3:** Comparison of the cross-cohort performances of models constructed based on clinical data and the integrated models.

Dataset	AUC	95% CI	Sensitivity	Accuracy
CRC				
ThomasAM_2018a	0·900	0·891–0·906	0·727	0·717
VogtmannE_2016	0·883	0·874–0·894	0·825	0·818
WirbelJ_2018	0·878	0·866–0·891	0·766	0·760
ZellerG_2014	0·924	0·916–0·932	0·826	0·826
CRA				
HanniganGD_2017	0·900	0·891–0·911	0·733	0·727
ThomasAM_2018a	0·867	0·854–0·879	0·833	0·818
YachidaS_2019	0·774	0·756–0·794	0·567	0·812
ZellerG_2014	0·889	0·878–0·901	0·694	0·714

### 3.5 Factors underlying the prediction of CRC/CRA

**FIGURE 6:**
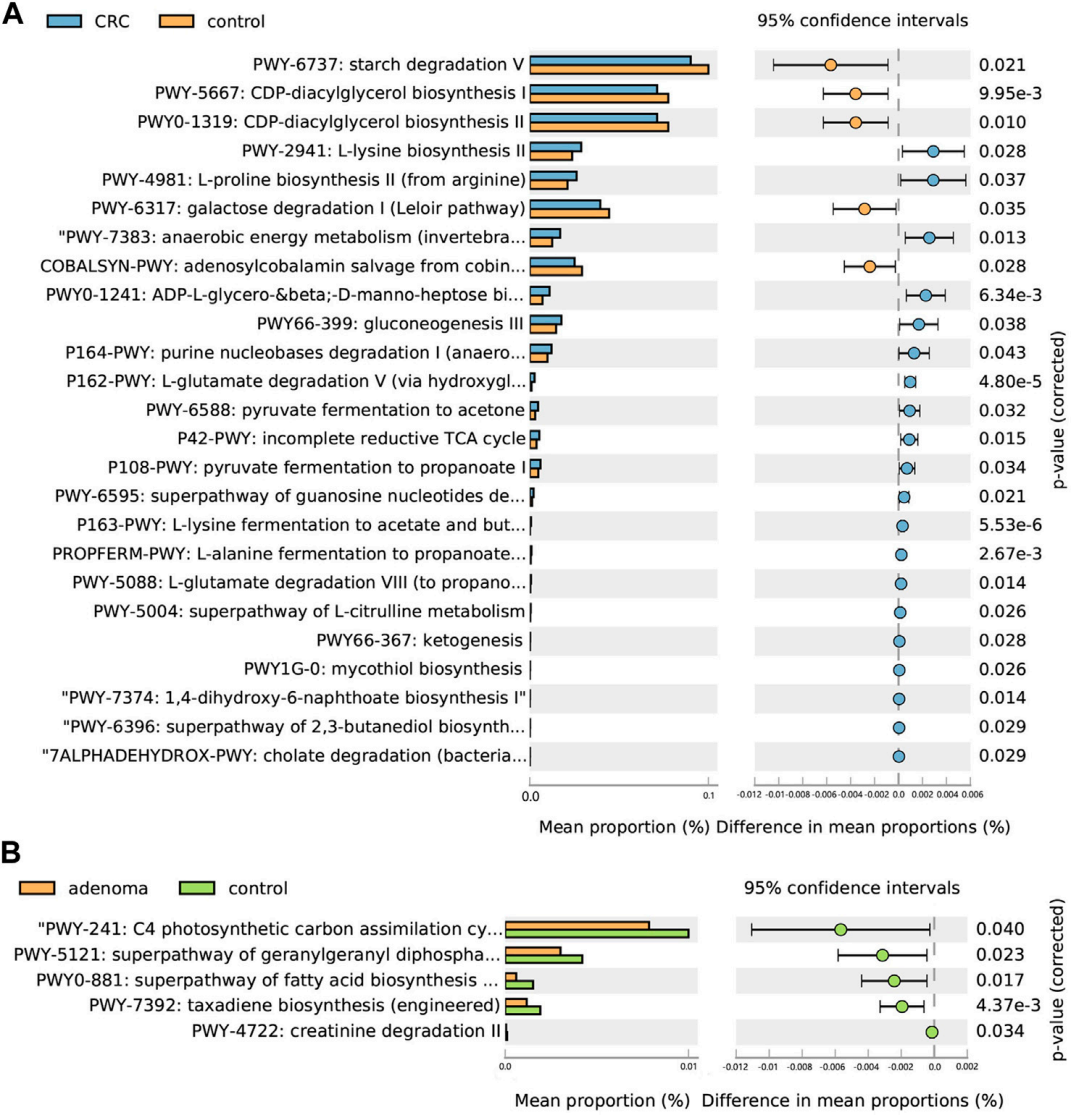
Differential functional pathways in CRC vs. HC **(A)** and CRA vs. HC **(B)**.

**FIGURE 7:**
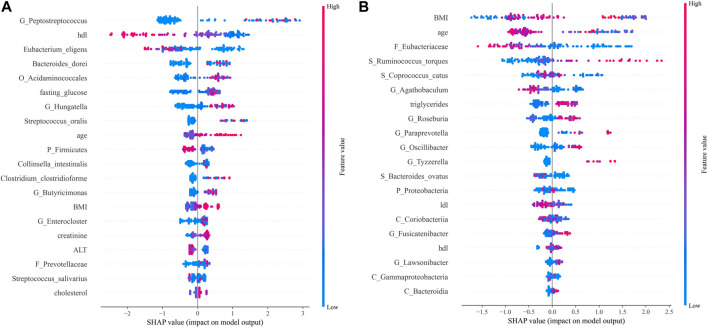
SHAP analysis results. Top 20 features of importance for prediction of CRC according to SHAP values in the integrated model **(A)**. Top 20 features of importance for prediction of CRA according to SHAP values in the integrated model **(B)**. Each dot in SHAP plot represents a patient, with x-axis location representing the SHAP value of the predicator. Dot color indicates the measured value of the predicator, assigned color red meant positive contribution and blue meant negative contribution. Features are ranked along the y-axis according to their contribution to model prediction.

Among the CRC and CRA top20 metagenomic feature groups. There is only one common metagenomic feature was identified, which is *Prevotellaceae* (Figure 6). We further performed separate differential analysis of functional pathways for CRC and CRA microbiome data, and the analysis showed that the differential pathways of CRC and CRA did not have any intersections (Figure 7).

**FIGURE 8:**
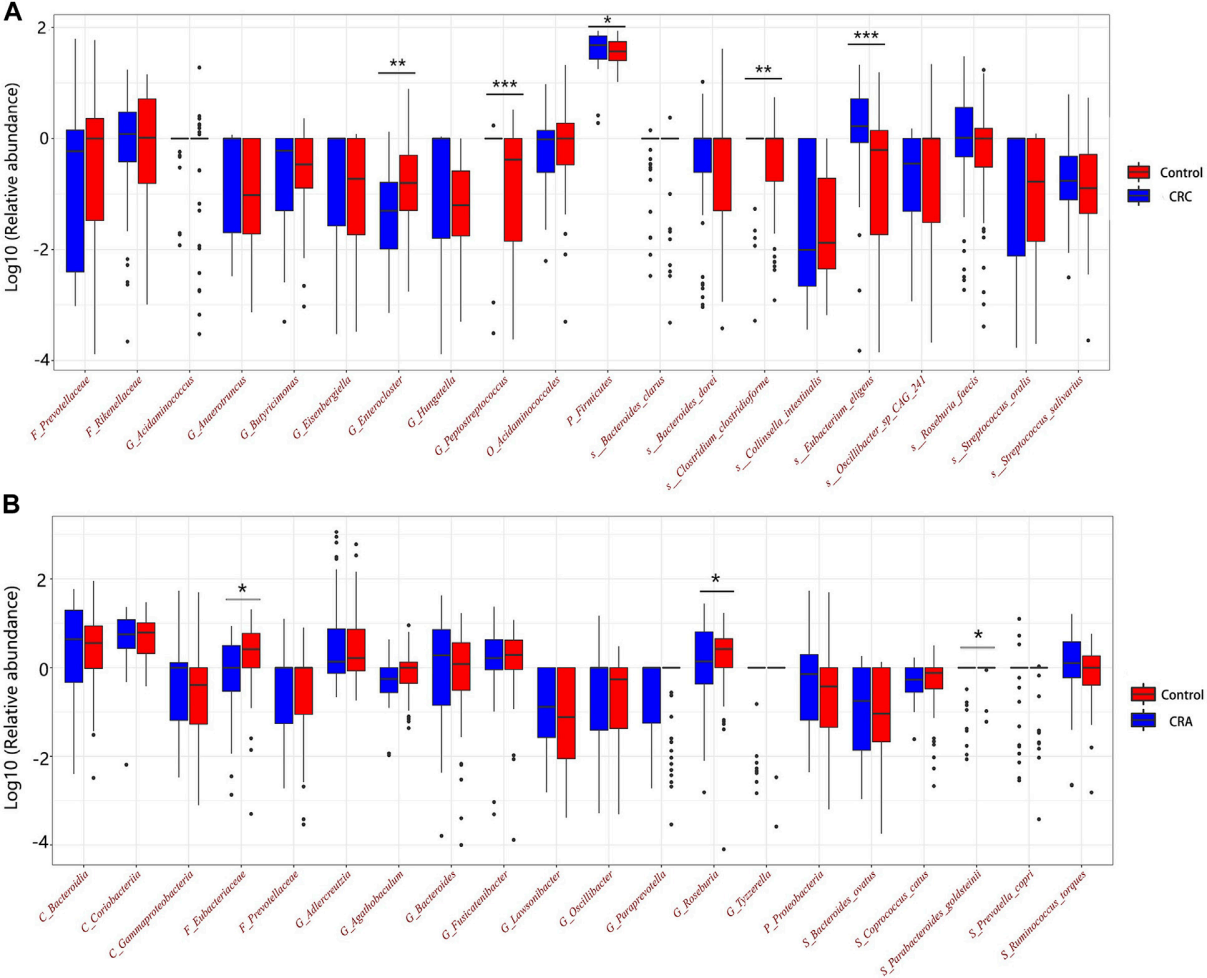
Relative abundances of the top 20 discriminative microbiota in CRC **(A)** and CRA **(B)**.

For CRC patients, the 5 most important features were *Peptostreptococcus*, HDL, *Eubacterium eligens*, *Bacteroides dorei*, and *Acidaminococcales*; among which HDL and *E. eligens* negatively contributed to CRC risk (Figure 8A).

**FIGURE 9:**
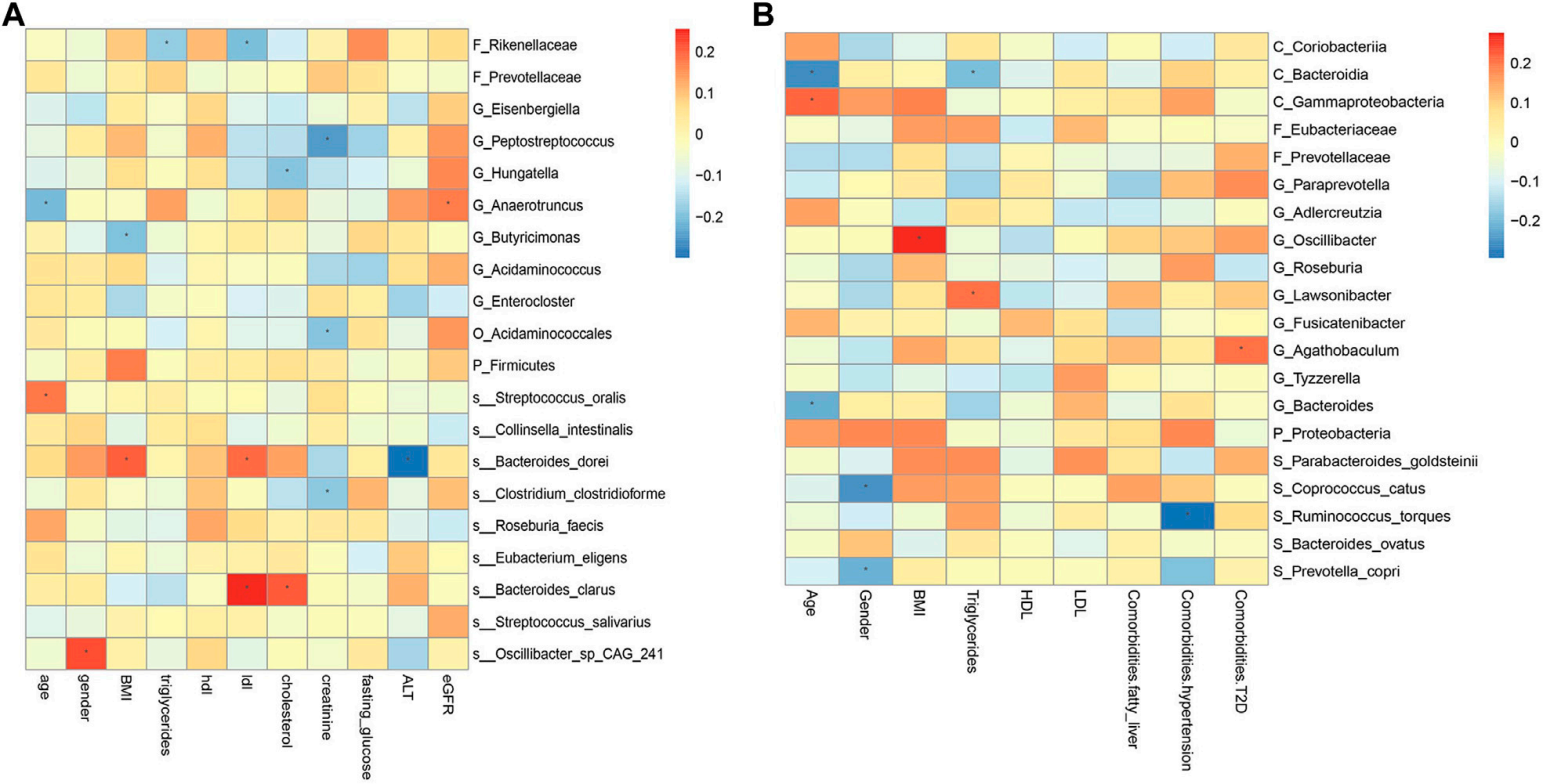
Spearman correlations between the abundances of top 20 discriminative gut microbiotaand clinical features in CRC **(A)** and CRA **(B)**. BMI, Body Mass Index; HDL, High-density lipoprotein; LDL, Low-density lipoprotein; ALT, Alanine aminotransferase; eGFR, estimated glomerular filtration rate. *, statistically significant after Bonferonni correction.

However, for CRA patients, the 5 most important features were BMI, age, low-density lipoprotein (LDL), Eubacteriaceae, *Ruminococcus torques*, and *Coprococcus catus*. Age, LDL, *Eubacteriacease, C. catus*, *Agathobaculum* and *Corobacteria* negatively contributed to CRA risk (Figure 8B). Correlations between gut microbiota and clinical features shown in Figure 9.

## 4 Discussion

In this study, we used gut metagenomic and clinical features to develop diagnostic models for CRC and CRA. Our model exhibited a better performance than clinically-used tests (e.g., gFOBTs, multi-target stool DNA, FIT, Methylated septin 9 gene, etc.) (Ladabaum et al., 2020). More importantly, we included data from 6 regions, including China, Germany, Italy, USA, Japan, and France, to address existing limitations regarding varied human gut microbiota compositions across populations with different environmental and genetic backgrounds. Through data mining optimization, feature selection, multi-omics analysis and other processes, stable CRC/CRA risk prediction models with generalizability were established, which could provide valuable insights for early CRC screening.

Several breakthroughs were made in this study. Firstly, previous disease diagnosis models based on gut microbiome nearly always performed poorly in external validations (Wong and Yu, 2019; Cammarota et al., 2020). However, we innovatively found that inputting full taxon data and adding cross-cohort tests simultaneously with features screening can help keep the balance between model performance and stability. Furthermore, the ML approach, instead of the traditional statistical models, is capable of taking the microbial community as a whole and determine the association between the structure of the community and the disease state (Handelman et al., 2018; Topçuoğlu et al., 2020; Stahlschmidt et al., 2022), yielding robust results. Secondly, we demonstrated that integrating clinical features in gut microbiome-based model significanlt improved model efficacy, especially for CRA. This holds clinical significance because accessing clinical indicators is convenient and inexpensive, and common features among regions could further enhance the stability and generalizability of model predictions. Furthermore, metagenomic features reflect the microenvironment of a localized lesion (Wong and Yu, 2019), while demographic and clinical features could reflect the overall disease states. A combination of these two aspects could provide a wholistic view of disease status, which could explain the reason that our integrated model outperformed existing models based only on microbiome in terms of stability and generalizability (Thomas et al., 2019; Wong and Yu, 2019).

Up to date, the current research on microbial integration models is still in its infancy, and most of the research focuses on integrating complex and expensive big data (e.g., exfoliated cells DNA sequencing data, microbe-associated metabolites, etc.), rather than obtaining convenient and affordable routine demographic and clinical data (Coker et al., 2022; Gao et al., 2022). However, this study showed that using clinical data for integration could exhibit comparable accuracy and sensitivity compared with other data. Compared with the model integrating metagenomic and metabolomics data by Coker and colleagues (Coker et al., 2022), our models’ efficacy was comparable to theirs regarding CRC diagnosis (Coker, AUC 95%CI: 91·5% −96·8%; This study, AUC 95%CI: 93·2%-94·7%), while outperforming the existing model regarding CRA diagnosis (Coker, AUC 95%CI: 83·6%-91·6%; This study, AUC 95%CI: 91·7%-93·5%).

Additionally, our results show that FOBT and clinical indicators related to lipid metabolism (HDL, LDL, etc.) were more critical for the diagnosis of CRC, while BMI and age contributed more to the CRA model.

*Prevotellaceae*, generally considered as probiotic for humans, was identified as the only overlapping microbial signature for CRC and CRA, and no overlapping functional pathway was found between the 2 disease states Figure 6. This suggests that CRC and CRA are two completely different disease states with different gut microbial environment, which is consistent with Casimiro-Soriguer’s view (Casimiro-Soriguer et al., 2022). However, there were certain feature similarities. For example, 10 taxonomic features found adversely contribute to CRC risk and 9 to CRA belonged to order *Clostridiales*, while 5 taxonomic features contributing to CRC risk and 6 to CRA belonged to order *Bacteroidales.* These findings are in line with a previous study (Baxter et al., 2014) that *Bacteroidales* is currently considered to be tumorigenic, while *Clostridiales* has been shown to be associated with colonic health. Hence, we suggest that in future clinical practices, monitoring changes in the levels of these two taxa could be beneficial for disease detection.

Moreover, nearly four-fifths of the top 20 features in the SHAP summary plot were from the metagenomic data, which could mean that the gut microbiome is crucial for the prediction of localized tumors or lesions such as CRC/CRA. The overabundance of *Peptostreptococcus* in CRC fecal samples has been found in multiple studies, and *Peptostreptococcus anaerobius* could enhance pro-inflammatory responses, cholesterol synthesis and cell proliferation (Karpiński et al., 2022). *Bacteroids dorei* was previously identidied as a CRC-infiltrating bacteria from a novel whole genome sequencing method of CRC tissue (Guo et al., 2019). Here, we can also infer that the dynamic between anti- and pro-inflammatory factors is crucial in CRC tumorigenesis, especially low-grade inflammation associated with metabolic disorders, as HDL (Rohatgi et al., 2021) and the anti-inflammatory species *E. eligens* negatively predicted CRC risk (Montilla and Villamiel, 2022), while increased abundance of *Acidaminococcales* was found in T2DM patients (Wang et al., 2020). BMI and age were the top 2 discriminating features identified in the diagnostic model for CRA, while Eubacteriaceae, generally considered to have anti-inflammatory properties (González-Mercado et al., 2020), protected against CRA. In conclusion, both microbiome and clinical features could help diagnose CRC and CRA.

The correlation between some metagenomic features and clinical indicators is also worthy of attention, which may suggest the mechanism behind the disease. For example, the correlation analysis results of YuJ_2017 data show (Figure 9A), LDL has a significant positive correlation with both *S_Bacteroides clarus* and *S_Bacteroides dorei* (*p* < 0.05). This may be related to the presence of cholesterol-reactive sulfotransferase in *Bacteroides bacteria* (Le et al., 2022). *G_Butyricimonas* is considered to be a beneficial bacteria that can improve human metabolism (Lee et al., 2022) and is inversely related to BMI. However, some bacterial species whose functions are not yet known to humans are also correlated with clinical indicators. For example, gender is significantly correlated with *S_Oscillibacter_sp_CAG_241* (*p* < 0.05). The results of this study also provide a direction for exploring bacterial species with unknown functions.

However, the main limitation of this study is that all data are from the publicly available databases, and the specificity of clinical data to CRC and CRA are dubious. Future modeling studies should adopt a prospective study design to include specific demographic and clinical risk factors to optimize prediction power. And due to the limitations of the current data, we lack time-series data to support our conclusions. In the future, we will work tirelessly to improve these issues.

In conclusion, we successfully constructed a cross-cohort and stable CRC and CRA diagnostic model integrating metagenomic and clinical features for early non-invasive screening of CRC and CRA. Compared with other CRC and CRA screening methods available, this model is more stable and generalizable. We also emphasize the importance of often overlooked demographic and clinical parameters in disease diagnosis and prediction models.

## Data Availability

The original contributions presented in the study are included in the article/Supplementary Material, further inquiries can be directed to the corresponding authors.
