# Supplementary material for: Integrating clinical and cross-cohort metagenomic features: a stable and non-invasive colorectal cancer and adenoma diagnostic model
Source: Front Mol Biosci. 2024 Jan 22;10:1298679. doi: 10.3389/fmolb.2023.1298679 (PMC10919151; doi:10.3389/fmolb.2023.1298679)
Supplement: Supplementary file 1 [file Table1.docx]

**Supplementary Table 1. Demographic and clinical characteristics of CRC and CRA patients and healthy controls.**

**Supplementary Table 1.1 Demographic and clinical characteristics of CRC (YuJ_2017) patients and healthy controls**

| **Clinical indicators** | **CRC**  **(N = 75)** | **Healthy people**  **(N = 53)** | **P-values** |
| --- | --- | --- | --- |
| **Age (Median, IQR)** | 67 (59, 74) | 64 (58, 65) | **0·012** |
| **Male (%, N)** | 64% (48/75) | 62.3% (33/53) | 0·988 |
| **BMI (Median, IQR)** | 23·0, (21·6, 24·8) | 23·8, (22·0, 25·9) | 0·151 |
| **Triglycerides (Median, IQR)** | 115·2, (79·7, 132·9) | 115·1, (72·6, 119·3) | **0**·**020** |
| **HDL (Median, IQR)^*^** | 50·3, (42·5, 58·0) | 51·7, (51·7, 73·5) | **0**·**001** |
| **LDL (Median, IQR)^*^** | 109·3, (88·9, 127·6) | 109·3, (96·7, 121·8) | 0·781 |
| **Cholesterol (Median, IQR)^*^** | 186·5, (162·4, 205·0) | 186·5, (175·9, 214·6) | 0·315 |
| **Creatinine (Median, IQR)** | 75·0, (63·0, 89·0) | 79·2, (66·5, 79·2) | 0·297 |
| **Fasting glucose (Median, IQR)** | 108, (95·4, 126·0) | 102·6, (90·0, 115·2) | **P<0**·**001** |
| **ALT (Median, IQR)** | 19, (13, 24) | 20·5, (19·0, 22·5) | 0·085 |
| **eGFR (Median, IQR)** | 71·8, (58·9, 88·7) | 73·9, (65·6, 77·0) | 0·799 |

*, Data were log-transformed to base 2.

**Supplementary Table 1.2 Demographic and clinical characteristics of CRA(FengQ_2015) patients and healthy controls**

| **Clinical indicators** | **CRA**  **(N = 47)** | **Healthy people**  **(N = 61)** | **P-values** |
| --- | --- | --- | --- |
| **Age (Median, IQR)** | 67 (62, 71) | 68 (65, 71) | 0·476 |
| **Male (%, N)** | 48·93% (23/47) | 59·0% (36/61) | 0·396 |
| **BMI (Median, IQR)** | 27·1, (24·4,30·5) | 29·0, (23·0, 30·4) | 0·293 |
| **Triglycerides (Median, IQR)** | 108, (79, 144) | 112, (84·5, 150) | 0·915 |
| **HDL (Median, IQR)** | 63·0, (51·0, 73·0) | 58·0, (47·0, 69·5) | 0·165 |
| **LDL (Median, IQR)** | 144·6, (117, 163) | 146, (116, 176) | 0·596 |
| **Comorbidities-T2D (%, N)** | 10·64% (5/47) | 21·31% (13/61) | 0·194 |
| **Comorbidities-hypertension (%, N)** | 53·19% (25/47) | 55·73% (34/61) | 0·945 |
| **Comorbidities-fatty liver (%, N)** | 59·57% (28/47) | 54·10% (33/61) | 0·709 |

**Supplementary Table 1.3 Demographic and clinical characteristics of CRC (ZellerG_2014) patients and healthy controls**

| **Clinical indicators** | **CRC**  **(N = 53)** | **Healthy people**  **(N = 61)** | **P-values** |
| --- | --- | --- | --- |
| **Age (Median, IQR)** | 67 (59, 74) | 63 (56, 67) | 0·007 |
| **Male (%, N)** | 48·93% (23/47) | 59·0% (36/61) | 0·384 |
| **FOBT(%,N)** | 49·1% (26/53) | 4·5% (3/67) | **0·001** |

**Supplementary Table 1.4 Demographic and clinical characteristics of CRC (VogtmannE_2016) patients and healthy controls**

| **Clinical indicators** | **CRC**  **(N = 52)** | **Healthy people**  **(N = 52)** | **P-values** |
| --- | --- | --- | --- |
| **Age (Median, IQR)** | 65, (54, 71) | 63, (55·75-68·25) | 0·800 |
| **Male (%, N)** | 71·1% (37/52) | 71·1% (37/52) | 1·000 |
| **BMI (Median, IQR)** | 24·33, (22·31-27·76) | 24·01, (23·00,27·31) | 0·589 |

**Supplementary Table 1.5 Demographic and clinical characteristics of CRC (ThomasAM_2018a) patients and healthy controls**

| **Clinical indicators** | **CRC**  **(N = 29)** | **Healthy people**  **(N = 24)** | **P-values** |
| --- | --- | --- | --- |
| **Age (Median, IQR)** | 71 (64, 71) | 67·5 (62·5, 71) | 0·103 |
| **Male (%, N)** | 79·3% (23/29) | 54·2% (13/24) | 0·051 |
| **BMI (Median, IQR)** | 24, (23, 26) | 25, (24,26·75) | 0·722 |

**Supplementary Table 1.6 Demographic and clinical characteristics of CRC (WirbelJ_2018) patients and healthy controls**

| **Clinical indicators** | **CRC**  **(N = 60)** | **Healthy people**  **(N = 65)** | **P-values** |
| --- | --- | --- | --- |
| **Age (Median, IQR)** | 63 (56,72) | 58 (49, 64) | **0·001** |
| **Male (%, N)** | 60% (36/60) | 56·9% (37/65) | 0·727 |
| **BMI (Median, IQR)** | 26·05, (23·75, 28·5) | 24·6, (22·8, 26·7) | **0·050** |

**Supplementary Table 1.7 Demographic and clinical characteristics of CRA (HanniganGD_2017) patients and healthy controls**

| **Clinical indicators** | **CRA**  **(N = 26)** | **Healthy people**  **(N = 28)** | **P-values** |
| --- | --- | --- | --- |
| **Age (Median, IQR)** | 63·5 (54·25, 68) | 54 (51, 61·5) | **0·025** |
| **Male (%, N)** | 57·7% (15/26) | 39·3% (11/28) | 0·176 |
| **BMI (Median, IQR)** | 26·7, (24·3,29·0) | 25·17, (21·46, 30·72) | 0·647 |
| **FOBT(%, N)** | 15·4% (4/26) | 0% (0/28) | **0·031** |

**Supplementary Table 1.8 Demographic and clinical characteristics of CRA (ThomasAM_2018a) patients and healthy controls**

| **Clinical indicators** | **CRA**  **(N = 27)** | **Healthy people**  **(N = 24)** | **P-values** |
| --- | --- | --- | --- |
| **Age (Mean, SD)** | 66·49±7·86 | 66·97±6·45 | 0·476 |
| **Male (%, N)** | 59·3% (16/27) | 54·2% (13/24) | 0·714 |
| **BMI (Median, IQR)** | 67·5 (62·5, 71) | 61 (57, 69) | **0·028** |

**Supplementary Table 1.9 Demographic and clinical characteristics of CRA (YachidaS_2019) patients and healthy controls**

| **Clinical indicators** | **CRA**  **(N = 67)** | **Healthy people**  **(N = 251)** | **P-values** |
| --- | --- | --- | --- |
| **Age (Median, IQR)** | 64, (58,71) | 63, (52,71) | 0.156 |
| **Male (%, N)** | 71.6% (48/67) | 54.2% (136/251) | **0.010** |
| **BMI (Median, IQR)** | 22.1, (20.29,24.74) | 22.36, (20.69,24.32) | 0.384 |

**Supplementary Table 1.10 Demographic and clinical characteristics of CRA (ZellerG_2014) patients and healthy controls**

| **Clinical indicators** | **CRA**  **(N = 42)** | **Healthy people**  **(N = 61)** | **P-values** |
| --- | --- | --- | --- |
| **Age (Median, IQR)** | 63 (59,68) | 63 (56, 67) | 0·262 |
| **Male (%, N)** | 71·4% (30/42) | 45·9% (28/61) | **0·010** |
| **FOBT(%, N)** | 11·9% (5/42) | 4·9% (3/61) | 0·345 |

P-values were calculated with Mann–Whitney U -test.
