# Supplementary material for: Integrating clinical and cross-cohort metagenomic features: a stable and non-invasive colorectal cancer and adenoma diagnostic model
Source: Front Mol Biosci. 2024 Jan 22;10:1298679. doi: 10.3389/fmolb.2023.1298679 (PMC10919151; doi:10.3389/fmolb.2023.1298679)
Supplement: Supplementary file 2 [file DataSheet3.docx]

**Supplementary Figure S4.**

**Spearman correlations between the abundances of top 20 discriminative gut microbiotaand clinical features** in CRC (A) and CRA (B). BMI, Body Mass Index; HDL, High-density lipoprotein; LDL, Low-density lipoprotein; ALT, Alanine aminotransferase; eGFR, estimated glomerular filtration rate.


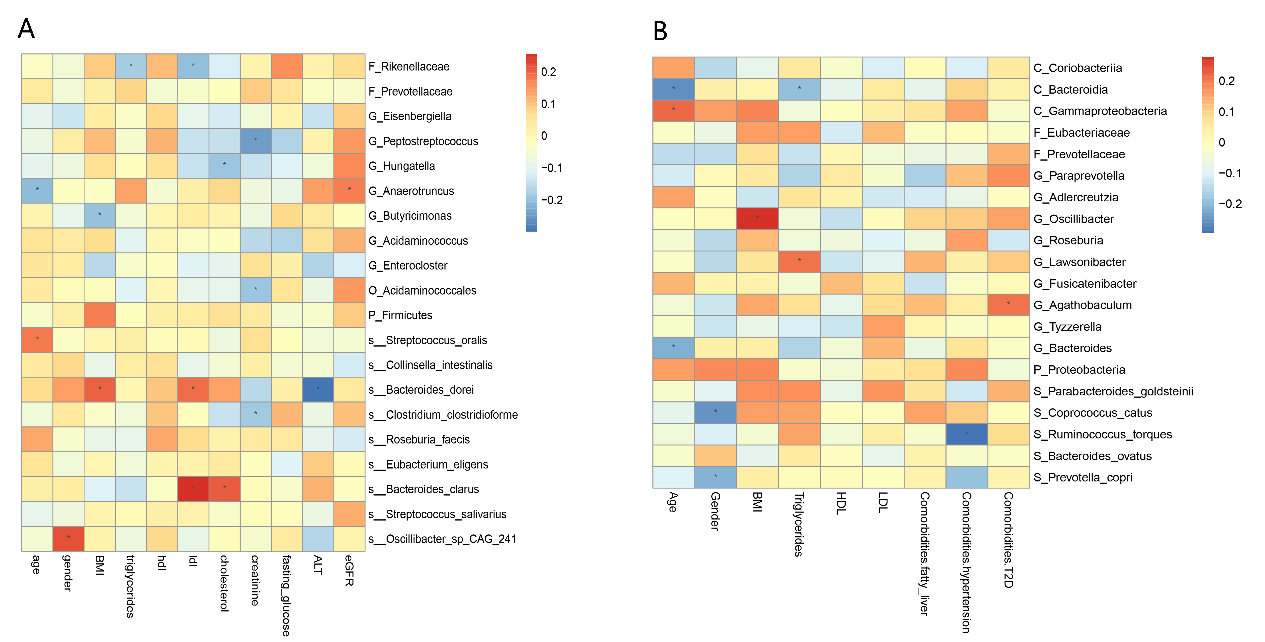


*, statistically significant after Bonferonni correction.
