# Supplementary figures and images for: Integrating clinical and cross-cohort metagenomic features: a stable and non-invasive colorectal cancer and adenoma diagnostic model
Source: Front Mol Biosci. 2024 Jan 22;10:1298679. doi: 10.3389/fmolb.2023.1298679 (PMC10919151; doi:10.3389/fmolb.2023.1298679)

**Supplementary Figure S3.**

**Differential functional pathways in CRC vs. HC (A) and CRA vs. HC (B).**


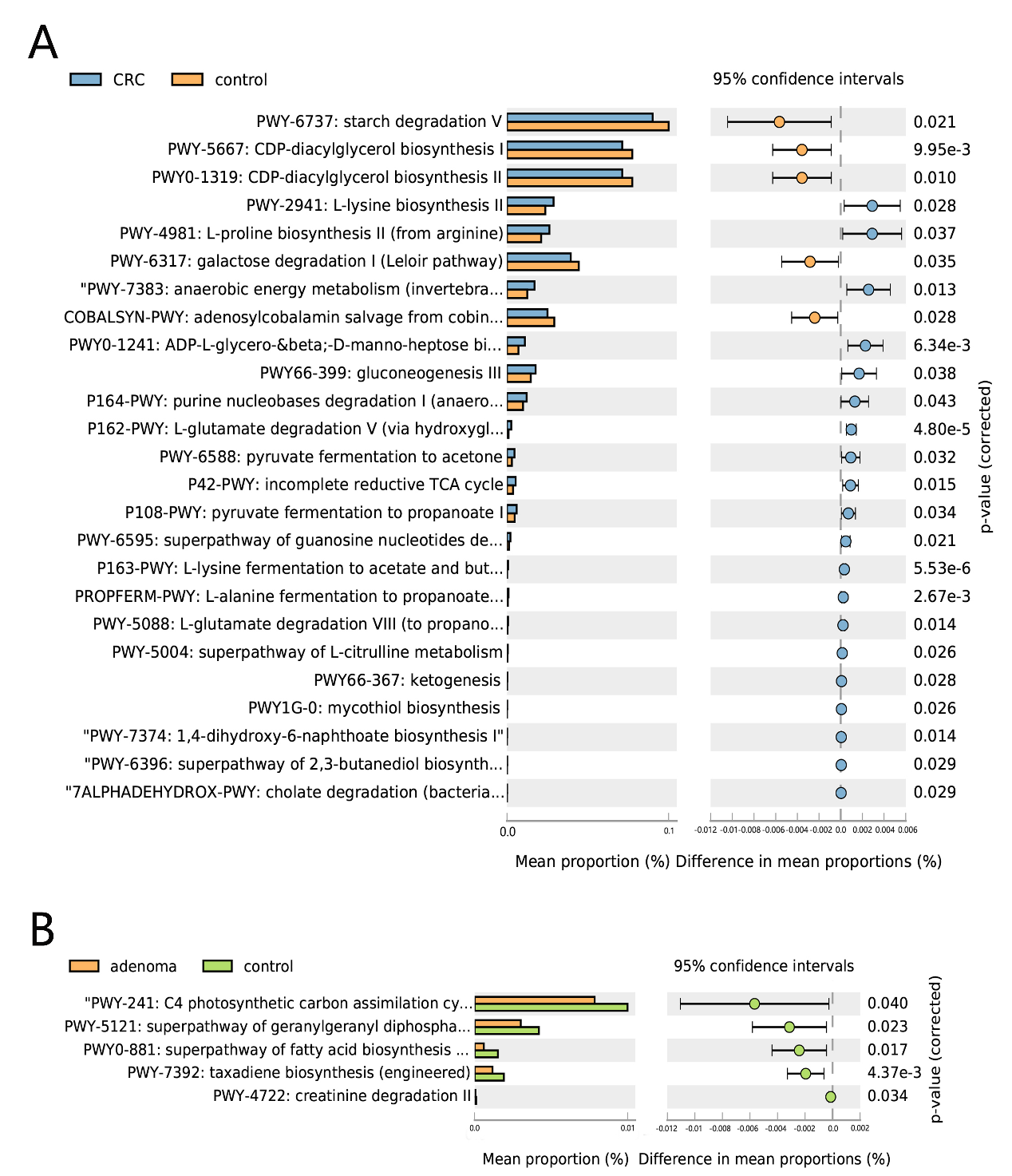

Supplement: Supplementary file 3 [file DataSheet2.docx]
