# Supplementary material for: Integrating clinical and cross-cohort metagenomic features: a stable and non-invasive colorectal cancer and adenoma diagnostic model
Source: Front Mol Biosci. 2024 Jan 22;10:1298679. doi: 10.3389/fmolb.2023.1298679 (PMC10919151; doi:10.3389/fmolb.2023.1298679)

**Supplementary Figure S2.**

**Relative abundances of the top 20 discriminative microbiota in CRC (A) and CRA (B).**


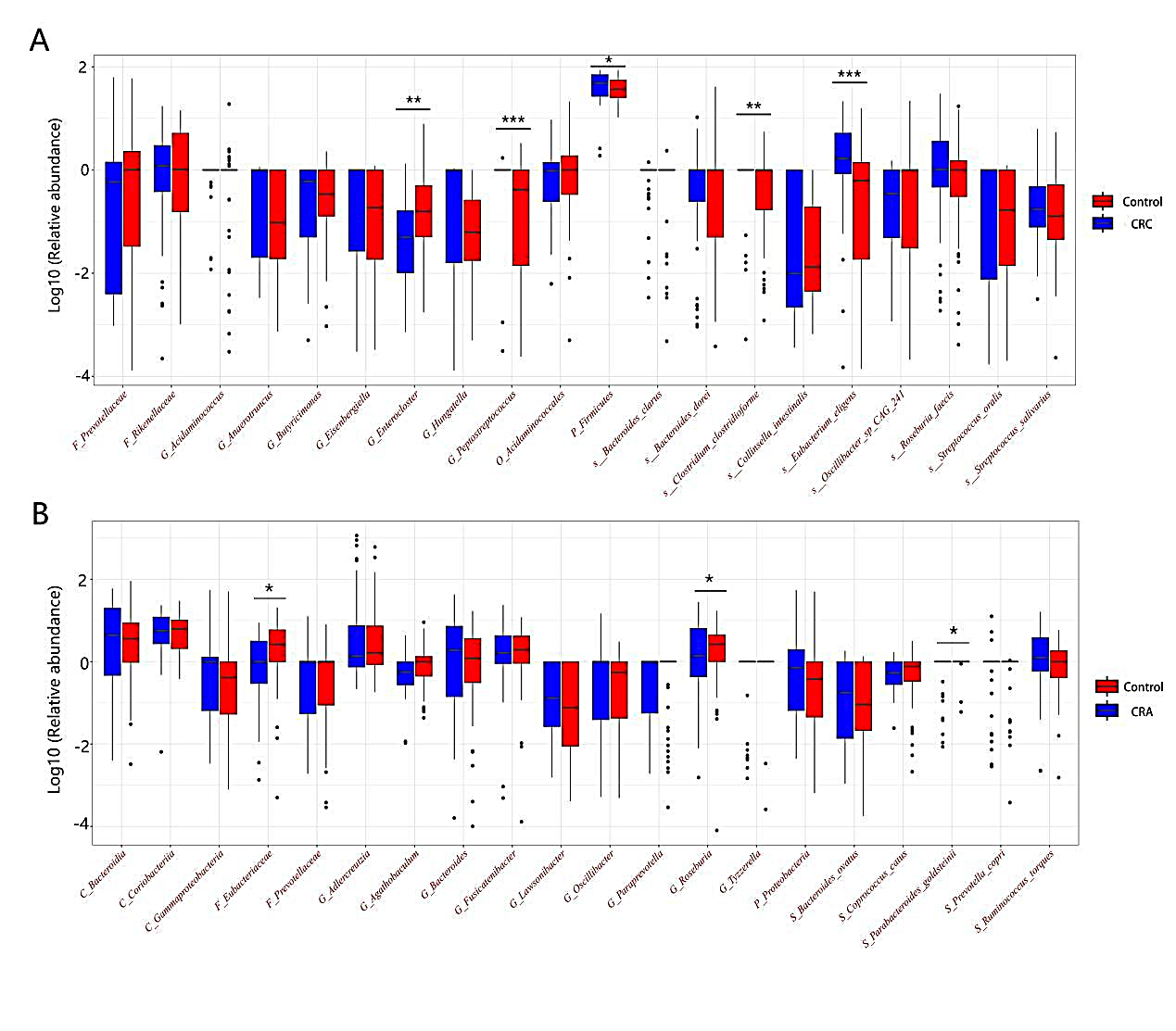

Supplement: Supplementary file 4 [file DataSheet1.docx]
