# Supplementary material for: Integrating clinical and cross-cohort metagenomic features: a stable and non-invasive colorectal cancer and adenoma diagnostic model
Source: Front Mol Biosci. 2024 Jan 22;10:1298679. doi: 10.3389/fmolb.2023.1298679 (PMC10919151; doi:10.3389/fmolb.2023.1298679)
Supplement: Supplementary file 5 [file Image1.pdf]

Supplementary Figure S1. Validation of the multi-modal machine learning models for diagnosis of CRC and CRA.

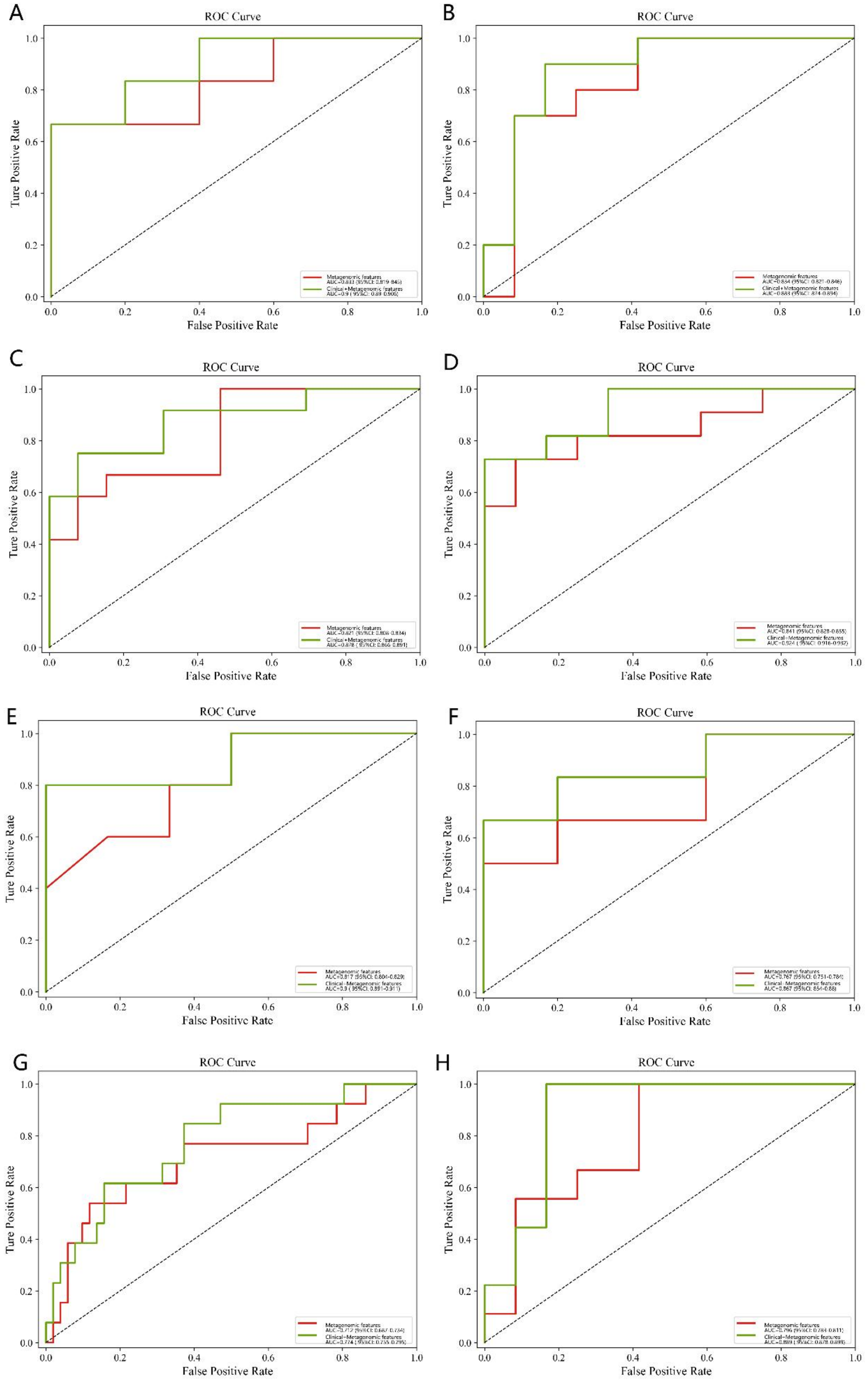

VogtmannE_2016 (USA, CRC), integrating clinical features of age, gender and BMI (A). ThomasAM_2018a (Italy, CRC), integrating clinical features of age, gender and BMI (B). WirbelJ_2018 (Germany, CRC) integrating clinical features of age, gender and BMI(C). ZellerG_2014 (France, CRC), integrating clinical features of age, gender and FOBT (D). HanniganGD_2017(China, CRA), integrating clinical features of age, gender, BMI and FOBT (E). ThomasAM_2018a(Italy, CRA), integrating clinical features of age, gender and BMI (F). YachidaS_2019(Japan, CRA), integrating clinical features of age, gender and BMI (G). ZellerG_2014 (France, CRA), integrating clinical features of age, gender, BMI and FOBT (H).
